# Manipulation on Two-Dimensional Amorphous Nanomaterials for Enhanced Electrochemical Energy Storage and Conversion

**DOI:** 10.3390/nano11123246

**Published:** 2021-11-29

**Authors:** Juzhe Liu, Rui Hao, Binbin Jia, Hewei Zhao, Lin Guo

**Affiliations:** 1 Beijing Advanced Innovation Center for Biomedical Engineering, Key Laboratory of Bio-Inspired Smart Interfacial Science and Technology, School of Chemistry, Beihang University, Beijing 100191, China; liujuzhe@buaa.edu.cn (J.L.); haorui628@buaa.edu.cn (R.H.); jiabinbin@buaa.edu.cn (B.J.); 2 School of Physics, Beihang University, Beijing 100191, China

**Keywords:** manipulation, two-dimension amorphous, component interaction, geometric configuration, electrochemistry

## Abstract

Low-carbon society is calling for advanced electrochemical energy storage and conversion systems and techniques, in which functional electrode materials are a core factor. As a new member of the material family, two-dimensional amorphous nanomaterials (2D ANMs) are booming gradually and show promising application prospects in electrochemical fields for extended specific surface area, abundant active sites, tunable electron states, and faster ion transport capacity. Specifically, their flexible structures provide significant adjustment room that allows readily and desirable modification. Recent advances have witnessed omnifarious manipulation means on 2D ANMs for enhanced electrochemical performance. Here, this review is devoted to collecting and summarizing the manipulation strategies of 2D ANMs in terms of component interaction and geometric configuration design, expecting to promote the controllable development of such a new class of nanomaterial. Our view covers the 2D ANMs applied in electrochemical fields, including battery, supercapacitor, and electrocatalysis, meanwhile we also clarify the relationship between manipulation manner and beneficial effect on electrochemical properties. Finally, we conclude the review with our personal insights and provide an outlook for more effective manipulation ways on functional and practical 2D ANMs.

## 1. Introduction

With the intensification of the global energy crisis, electrochemical energy storage and transformation has become one of the most concerned research hotspots in the world. Therefore, it is necessary to develop efficient, clean, and sustainable energy technologies, such as supercapacitor, battery, and electrocatalysis. As the core parts of these systems, electrode materials have experienced vigorous development and achieved multi-size, multi-dimensional, and multi-component precise regulation to adapt the diverse and complex energy storage and transformation processes [1,2,3,4].

Electrochemical performance is closely related to the structure of electrode materials. The two-dimensionalization of electrode materials can increase electrochemically active surface area (ECSA) and facilitate ion diffusion for enhanced electrochemical performance, which has drawn extensive attention [5,6,7,8]. Different from conventional material control strategies mainly concentrated upon composition, morphology, and dimension, crystal phase control demonstrates some superiority, especially for enhancing performance. Many materials have more than one phase, which is mainly determined by chemical bonds and thermodynamic parameters. By precisely controlling various structural parameters, it is possible to obtain non-thermodynamically stable phase structure with disordered atomic arrangement over a long range and only short-range order over a few atoms, that is amorphous structure. The materials with amorphous structure are isotropic, lack grain boundaries, and endowed with inherent abundant defects, which have come into people’s attention and worked as advanced electrode materials [9]. For instance, Lei et al. found amorphous titanium dioxide to be an efficient electrode material for sodium ion batteries with impressive charge storage capacity and cycle life [10]. Therefore, it is challenging but meaningful to combine the merits of two-dimensional and amorphous structures for developing well-performed, two-dimensional amorphous nanomaterials (2D ANMs). Compared with conventional materials, 2D ANMs generally exhibit distinctive features: i) ultra-high specific surface area and plentiful defects, which can provide more exposed active sites; ii) favorable diffusion paths and distances, which are conductive to the insertion/extraction of reactants and products; iii) strong in-plane covalent bond and lacking of grain boundary enhancing mechanical properties for extended volume or shape change; iv) flexible morphology and composition providing an additional degree of freedom for further modification; v) unprecedented electron state induced by confinement of electrons in 2D scale, which may facilitate electron transfer and electrode reactions.

Up to now, many 2D ANMs have been synthesized and applied in energy storage and transformation processes, including carbon materials [11], black phosphorus [12], metal compounds [13,14,15,16], etc. For example, Guo et al. developed amorphous cobalt-vanadium hydr(oxy)oxide nanosheets as an efficient electrocatalytic material for oxygen evolution reaction (OER) superior to the crystalline counterparts [17]; Wu and co-workers prepared 2D amorphous Cr_2_O_3_/graphene nanosheets by rapidly heating hydrous chlorides, which exhibited ultrahigh reversible capacity and outstanding cycle life in Li-ion battery outperforming crystalline nanoparticles [18]. However, the development of 2D ANMs is relatively lagging for their immature synthetic methods and drawbacks in application, such as low conductivity and instability. On the face of it, some strategies have been proposed to manipulate 2D ANMs based on their flexible structures and compositions for enhanced electrochemical energy storage and conversion [19,20]. This review aims at illuminating the modes and roles of manipulating 2D ANMs in electrochemical fields (supercapacitor, battery, and electrocatalysis) in terms of geometric configuration design and component interaction. First, we pay attention to manipulation strategy of 2D ANMs in recent years. Following this, we emphatically discuss their application and electrochemical mechanism. Finally, we conclude our personal insights and provide outlook for the development of 2D manipulative amorphous nanomaterials. We hope that the integration of 2D manipulative amorphous nanomaterials in electrochemistry will offer great opportunities to address the challenges driven by the increasing global electrochemical energy storage and transformation processes.

## 2. Manipulation Strategy of 2D ANMs

### 2.1. Synthesis Methods

**Figure 1:**
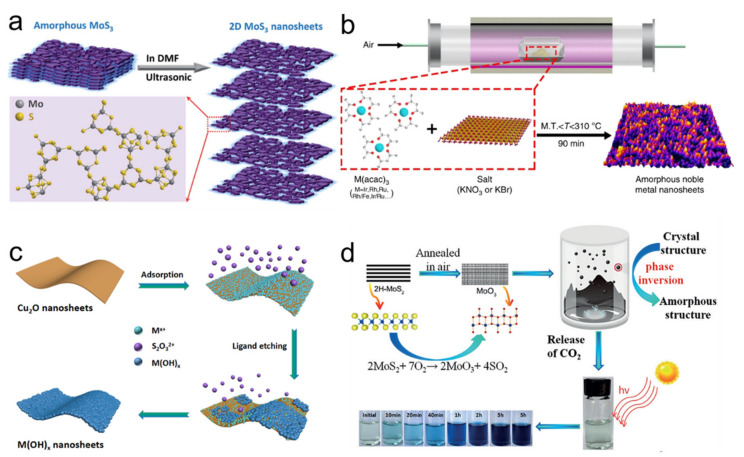
(**a**) Schematic illustration of exfoliation 2D amorphousMoS_3_ nanosheets and their chemical structure. Reprinted with permission from Ref. [23]. Copyright Royal Society of Chemistry, 2019. (**b**) Schematic illustration of the general synthetic process for amorphous noble metal nanosheets. Reprinted with permission from Ref. [33]. (**c**) The schematic illustration of the synthesis of amorphous metal hydroxide nanosheets. Reprinted with permission from Ref. [28]. Copyright Royal Society of Chemistry, 2019. (**d**) Schematic illustration of the formation mechanism for amorphous MoO_3_ nanosheets. Reprinted with permission from Ref. [31]. Copyright Wiley-VCH, 2017.

J. Kotakoski et al. firstly used electron irradiation to create 2D amorphous carbon material in 2011, which opened new possibilities for preparing 2D ANMs [21]. For some intrinsic bulk materials that are amorphous nanomaterials, the desired 2D ANMs can be obtained by exfoliation. For example, dimethylformamide (DMF) is an ideal solvent to exfoliate bulk MoS_2_ relative to other solvents due to its low surface tension (~40 mJ m^−2^) [22]. In view of this fact, 2D amorphous MoS_3_ nanosheets can be successfully obtained by the exfoliation of the bulk amorphous MoS_3_ material in DMF solvent under ultrasonic irradiation (Figure 1a) [23]. Other types of 2D ANMs have been obtained by many reliable methods including thermal decomposition [24], electrodeposition [25,26], template method [27,28,29], phase transformation [30,31], and element doping [17,32]. Synthesis of amorphous noble metal nanostructures is always a great challenge for their strong and isotropic nature of metallic bonds. In view of this, Li et al. proposed a simple method for preparing amorphous noble metal nanosheets by directly annealing metal acetylacetonate with alkali salt (Figure 1b) [33]. The synthesis temperature was between the melting point of metal acetylacetone and the melting point of alkali salt. When alkali salt was removed by deionized water, high yield amorphous noble metal nanomaterials can be successfully obtained, including monometal nanosheets, bimetal nanosheets, and trimetal nanosheets. Guo et al. utilized sacrificial template strategy to yield a library of ten distinct 2D ultrathin amorphous metal hydroxide nanosheets [28]. The key point of the synthesis is based on the balance between the etching rate of the Cu_2_O template and deposition rate of the metal hydroxide. As shown in Figure 1c, Cu_2_O was first employed as a sacrificial template to promote the 2D planar growth of metal hydroxides into a nanosheet structure. Then, S_2_O_3_^2−^ can react with Cu_2_O to produce OH^−^ ions. Finally, after the concentrations of OH^−^ ions increased to the precipitation threshold, metal ions could combine with OH^−^ to form 2D amorphous sheet structure. In general, most of them are based on the classical 2D crystalline nanomaterials synthetic theory by introducing some mechanisms of inhibiting crystallization. The common inhibition factors involve shorting reaction time, reducing reaction temperature, destroying crystal structure, etc.

It needs to be clarified that some target amorphous products are difficult to prepare by one synthesis method and other methods should be involved. Xu et al. combined the oxidation of MoS_2_ and supercritical CO_2_ treatment strategy to prepare amorphous molybdenum oxide (MoO_3_) nanosheets [31]. As shown in Figure 1d, single-layer or few layers of crystalline MoS_2_ were firstly exfoliated. Then, oxygen atoms replaced sulfur atoms to destroy the regular atomic arrangement of MoS_2_ during the annealing process. Finally, the stable amorphous MoO_3_ was obtained by the adsorption of CO_2_.

### 2.2. Manipulation Modes

2D amorphous material has flexible structure and composition that allows dexterous manipulation. As mentioned above, various strategies have been proposed to manipulate 2D ANMs for enhanced electrochemical performance. We generalize and conceptualize these strategies to be two major categories of geometric configuration design and component interaction. According to our understanding, the geometric configuration design mainly includes spatial structure design at micro/nano scale and coordination environment design at atomic scale. The component interaction mainly includes elemental interaction and heterophase compositing. Here, the relevant enhanced effects and implementation approaches will be introduced.

#### 2.2.1. Geometric Configuration Design

##### Spatial Structure Design

**Figure 2:**
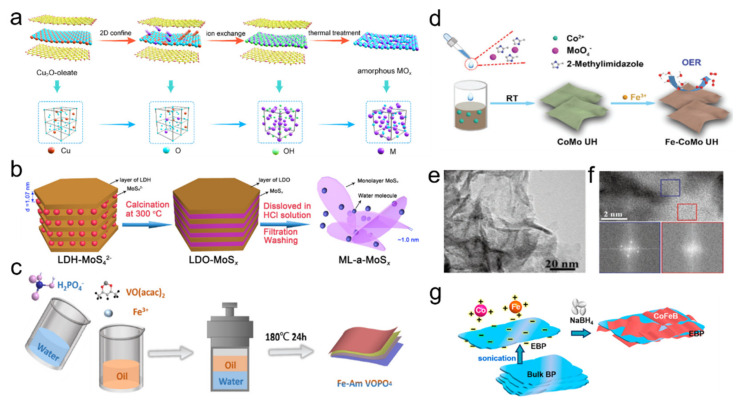
(**a**) Schematic illustration for synthesis of amorphous metal oxide ultrathin nanosheets. Reprinted with permission from Ref. [35]. Copyright American Chemical Society, 2020. (**b**) Schematic of the preparation process for amorphous MoS_x_ monolayer nanosheets with abundant Mo defects. Reprinted with permission from Ref. [37]. Copyright Elsevier, 2020. (**c**) Schematic representation of the one-pot preparation processes of Fe-doped amorphous VOPO_4_. Reprinted with permission from Ref. [38]. Copyright Elsevier, 2021. (**d**) Schematic illustration of amorphous Fe-doped CoMo ultrathin hydroxide nanosheets. Reprinted with permission from Ref. [39]. Copyright Royal Society of Chemistry, 2021. (**e**) HRTEM image of CoV-Fe hydroxide nanosheets and (**f**) corresponding FFT patterns of selected regions marked by blue and red squares, respectively. Reprinted with permission from Ref. [40]. Copyright Wiley-VCH, 2020. (**g**) Schematic illustrations of the synthesis of EBP/CoFeB nanosheets. Reprinted with permission from Ref. [41]. Copyright American Chemical Society, 2021.

Spatial structure design at micro/nano scale can be deemed as the manipulation on the shape, size, packing form, and porous structure of 2D ANMs, which can be controlled by template design and reactive conditions [34]. Specifically, endowing 2D ANMs with porous structure should be an advisable way for enhanced electrochemical property. In electrocatalysis process, porous nanostructure can provide large surface area and abundant active sites, ensure effective penetration of electrolyte ions and escape of products, and alleviate stacking problem of nanosheets. As a typical case for creating pores on 2D ANMs, Guo group proposed a universal strategy combining confined method and ion exchange strategy to synthesize a series of 2D porous amorphous metal oxide nanosheets, such as Fe_2_O_3_, Cr_2_O_3_, ZrO_2_, SnO_2_, and Al_2_O_3_ [35]. The schematic illustration for synthesis of ultrathin amorphous metal oxide nanosheets was demonstrated in Figure 2a. Firstly, lamellar oleate was introduced as a host matrice to restrict the Cu_2_O template. Secondly, the target metal ion was replaced by Cu^+^ ions and introduced into 2D space through ion exchange strategy to form corresponding amorphous M(OH)_x_-oleate complex precursor. Finally, porous structure and disorder atom arrangement were achieved for metal oxide product by removing oleate and hydrone in heat treatment.

##### Coordination Environment Design

Tuning coordination environment at atomic scale can change the state of active sites, which afford improved electrochemical efficiency. Defect design is the most commonly used way and atomic-scale defects can be classified as anion vacancy, cation vacancy, associated vacancy, pits, distortions, and disorder [36]. Creating defects is generally deemed to be conductive to the mobility and adsorption of reactants and optimize reactive energy paths. In contrast to crystalline materials, precise design, and identification on defects are relatively difficult for amorphous ones with disorder atomic structure assembling massive and various defects, especially for 2D ANMs. Nonetheless, some efforts have been devoted to defect manipulation on 2D ANMs. Selective component removal or addition should be an effective way. Typically, Hou et al. developed amorphous MoS_x_ monolayer nanosheets with abundant Mo defects using the space-confined strategy [37]. The synthesis details are shown in Figure 2b. The precursor of layered double hydroxide with MoS_4_^2−^ (LDH-MoS_4_^2−^) was first synthesized via dispersing a layered double oxide (LDO) in an aqueous solution of (NH_4_)_2_MoS_4_. Afterwards, the obtained precursor was calcined in a N_2_ atmosphere to form amorphous MoS_x_ monolayer nanosheets in the interlayer space of LDO. Finally, amorphous MoS_x_ monolayer nanosheets were successfully obtained by washing in a nonoxidative HCl solution to dissolve LDO (the host layers). In this process, the generation of Mo defects can be adjusted by calcination temperature, which affects the S/Mo atomic ratio.

#### 2.2.2. Component Interaction

##### Elemental Interaction

Doping or coupling other elements may be a feasible method to enhance the electrochemical performance of 2D ANMs due to the multielement synergy effect. Commonly adopted strategies are direct coupling and post-doping. Wei et al. fabricated Fe-doped amorphous VOPO_4_ in solvothermal environment by one-pot two-phase colloidal method (Figure 2c) [38]. The oil phase consisted of oleylamine (OM) and octadecene (ODE) dissolved with Fe and V precursor, which is mixed with water phase containing sodium dihydrogen phosphate, and then was sealed in an autoclave and heated to get the final product. Figure 2d demonstrated a typical post-doping way that crystalline CoMo ultrathin hydroxide was firstly constructed by coprecipitation reaction and then amorphous Fe-doped CoMo ultrathin hydroxide nanosheets was obtained by ion exchange process [39].

##### Heterophase Compositing

Compositing is a common means to combine a different phase with 2D ANMs. It can integrate advantages and realize optimized design on interfacial structure, holistic architecture and physical property, embody in enhanced conductivity, modulated electron structure and active sites, improved stability, etc. Recently, the introduction of crystal phase into the amorphous phase to form a crystalline/amorphous hybrid dual-phase structure has attracted much attention, due to the unique properties produced by the phase boundary. The flexible amorphous structure has abundant active centers, which can enhance the electrochemical activity, while the crystalline structure possesses a highly symmetrical nonflexible structure, which can enhance the stability of the material. Yan et al. prepared hybrid dual-phase materials by doping Fe in CoV hydroxide nanosheets composed of a large number of crystalline and amorphous domain mixtures (Figure 2e,f) [40].

The unique interfaces of the catalyst promote the exposure of the active center, adjust the local coordination environment and electronic structure, and reduce the thermodynamic barrier during the OER catalytic reaction. Carbon materials are desirable candidates to form compositing structure with 2D ANMs. Wen et al. reported an exfoliated black phosphorus/CoFeB nanosheet (EBP/CoFeB) implemented by three steps under a N_2_ atmosphere (Figure 2g) [41]. First, EBP was obtained from bulk phosphorus by liquid stripping; then metal ions (Co^2+^, Fe^2+^) were adsorbed on the surface of EBP through electrostatic interaction; finally, CoFeB nanosheets were grown on EBP through chemical reduction initiated by NaBH_4_. Both of them provide good demonstration on manipulating 2D ANMs by compositing way. Besides, many amorphous nanosheets deposited on various conductive substrates have been successfully synthesized to enhance the electrochemical performance, such as nickel foam [42,43], graphite [44,45], graphene [27], and TiO_2_ mesh [46].

## 3. Manipulating 2D ANMs for Batteries and Supercapacitors

Well-manipulated 2D ANMs are attractive and show considerable application potential for diverse electrochemical systems, benefiting from their unique properties including abundant pores for ion storage capacitance, larger interlayer distance for ion de/intercalation, enhanced conductivity and elemental interaction by compositing. In this section, we mainly concentrate on introducing the manipulative strategies on 2D ANMs in renewable energy technologies including rechargeable battery (Lithium-ion battery (LIB), Sodium-ion battery (SIB), Potassium-ion battery (KIB)) and Supercapacitor (SC).

### 3.1. Rechargeable Battery

**Figure 3:**
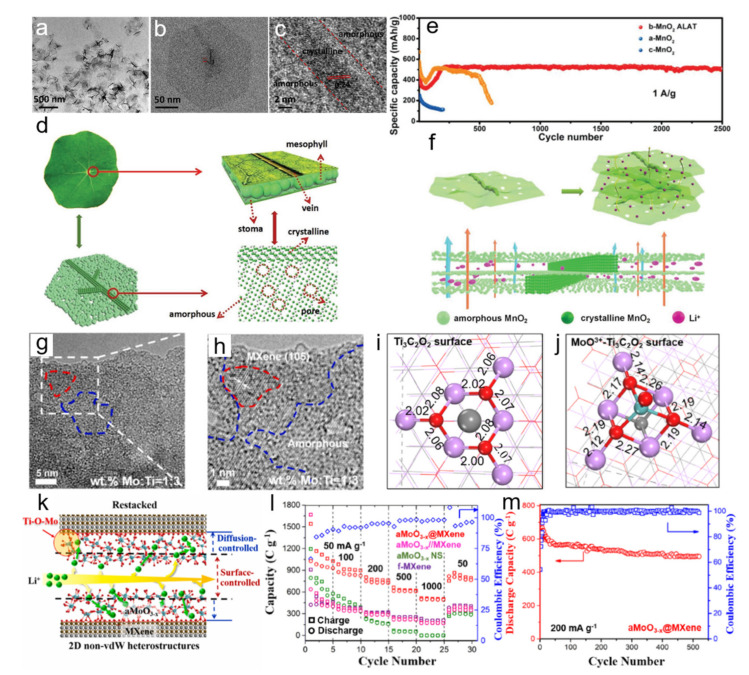
(**a**,**b**) TEM images of b-MnO_2_ ALAT; (**c**) HRTEM image of b-MnO_2_ ALAT; (**d**) Illustration of the breathable artificial leaves structure; (**e**) Cycling performance of b-MnO_2_ ALAT at 1 A g^−1^; (**f**) Schematic illustration of advantageous features of b-MnO_2_ ALAT for energy storage. Reprinted with permission from Ref. [48]. Copyright Wiley-VCH, 2019. (**g**,**h**) HRTEM images of 2D heterostructures of aMoO_3-x_@MXene non-vdW heterostructures; (**i**,**j**) Ti-O bond lengths in MXenes before and after the adsorption of MoO^3+^, respectively; (**k**) Illustration of facile capacitor-like interlayer diffusion and diffusion-controlled interlayer diffusion; (**l**) Cycling performance at different rates for aMoO_3-x_ NS, self-assembled aMoO_3-x_//MXene vdW heterostructures, and aMoO_3-x_@MXene non-vdW heterostructures; (**m**) Cycling performance for aMoO_3-x_@MXene non-vdW heterostructures at 200 mA g^−1^. Reprinted with permission from Ref. [49]. Copyright Elsevier, 2021.

Geometric configuration is an effective way to operate 2D ANM in order to overcome the obstacles of volume expansion and faded capacity for electrode materials in long cycles [47]. Specifically, by introducing heterostructure, the electrochemical cycle-life and rate performance should be apparently improved. Guo group reported a breathable 2D MnO_2_ artificial leaf with atomic thickness (b-MnO_2_ ALAT) and proposed the manipulation approach of 2D ANMs by modifying the pore structure and compositing crystalline skeleton on the amorphous substrate (Figure 3a–c) [48]. This obtained ultrathin leaf-like structure comprises of amorphous microporous mesophyll-like nanosheet as substrate and vein-like crystalline skeleton as support (Figure 3d). As shown in Figure 3e, when used as the anode material for LIBs, it delivered high capacity of 520 mAh g^−1^ and extremely stable cycle life over 2500 cycles at 1.0 A g^−1^, overcoming the disadvantages of pure MnO_2_ with irreversible capacity loss and poor cycling behavior. The outstanding electrochemical performance was elaborated as follows (Figure 3f): First, 2D nanostructure possessed large surface area, which can accommodate the volume changes associated with electrochemical reactions; Second, porous amorphous structure guaranteed the effective wetting and penetration of electrolyte, offered continuous charge transport pathway, and buffered volume changes and shortened ion diffusion distance. Third, the vein-like crystalline support could perfectly solve the closely stacking problem of 2D ultrathin nanosheets since it could leave a small inter space between overlapped nanosheets and effectively increase the number of lithium-storage sites and ion diffusion rate.

Based on the same strategy, Xu et al. explored a novel non-van der Waals (non-vdW) heterostructure of 2D amorphous MoO_3-x_ (aMoO_3-x_) nanosheet on Ti_3_C_2_-MXene (Figure 3g,h), which displayed superior electrochemical properties than counterparts. Density functional theory (DFT) calculations (Figure 3i,j) suggested that the amorphous non-vdW heterostructure can strongly stabilize aMoO_3-x_ nanosheet contributing to the improved stability and conductivity as well as facile Li ion diffusion. The restacked 2D heterostructures provide additional 2D Li^+^ diffusion pathways (Figure 3k), where a significant amount of Li^+^ can be stored on the surface defects and surface vacant sites of aMoO_3-x_. When applied as the anode for LIB, it shows excellent rate capability (Figure 3l) and high reversible capacity (Figure 3m), which is more outstanding than those of self-assembled aMoO_3-x_/MXene vdW heterostructure and bulk aMoO_3-x_ [49]. Huang et al. integrated 2D porous amorphous Si nanoflakes with ultralong multiwalled carbon nanotubes (MWCNTs) as a freestanding film electrode with high volumetric capacity and energy density. The interconnected network can prevent adjacent amorphous nanoflakes from restacking and the 2D porous structure provides large electrode/electrolyte contact area, both of which can enhance fast Li^+^ transportation and suppress the volume change [50]. Wang et al. successfully composited amorphous MoS_2_ with different carbon-based nanomaterials as LIB anode for increased conductivity and energy density [51].

**Figure 4:**
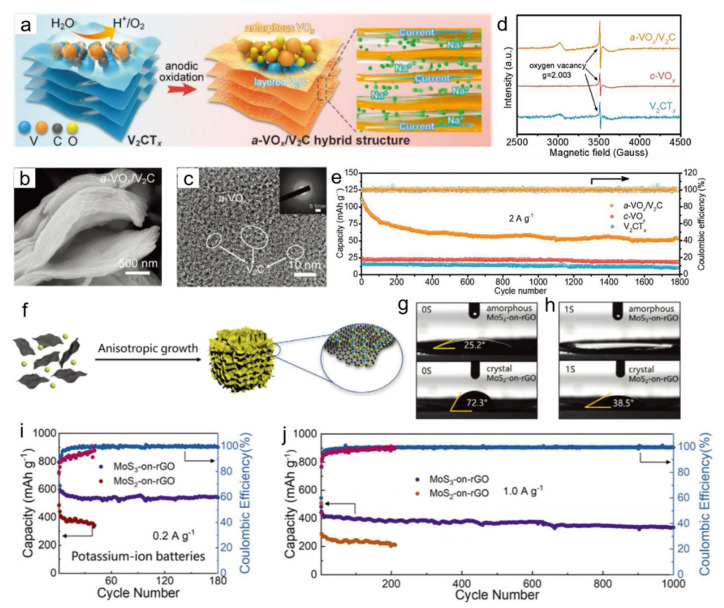
(**a**) Schematic illustration showing the synthesis and structure; (**b**) SEM image; (**c**) HRTEM image, the inset is the SAED pattern; (**d**) EPR spectra; (**e**) Cycling performances of a-VO_x_/V_2_C. Reprinted with permission from Ref. [52]. Copyright Wiley-VCH, 2021. (**f**) Schematic illustration of growth behaviors of amorphous MoS_3_-on-rGO; (**g**,**h**) Contact angle images of electrolyte on the electrode surface of amorphous MoS_3_-on-rGO and crystal MoS_2_-on-rGO. (**i**) Cyclic performance of KIB at 0.2 A g^−1^. (**j**) Long cycle performance of KIB at 1.0 A g^−1^. Reprinted with permission from Ref. [53]. Copyright Wiley-VCH, 2021.

Component interaction is another satisfactory approach to further optimize the relationship of 2D amorphous structure and electrochemical properties. SIB and KIB are considered to replace LIB and become the protagonist of the next generation of energy storage. However, the ion de-intercalation and volume expansion problems caused by the large ion radius of sodium and potassium are still serious obstacles in the actual application process. Hence, adaptable 2D ANMs should be ideal candidates for SIB and KIB, benefiting from synergistic effects including improved conductivity, enlarged interlayer, reformed wettability and introduced vacancies. Wang et al. successfully explored a nanohybrid of amorphous vanadium oxide on V_2_C MXene (a-VO_x_/V_2_C) (Figure 4a). The coexistence of a-VO_x_ and V_2_C is demonstrated in Figure 4b,c. Electron paramagnetic resonance revealed that the a-VO_x_/V_2_C electrode with disordered V–O framework generated more oxygen vacancies than c-VO_x_ (Figure 4d), which would favor fast Na^+^ insertion/extraction. When used as the anode for SIBs, it possesses more excellent cycling performance compared to the c-VO_x_ (Figure 4e) [52]. Yu and co-workers prepared 2D amorphous MoS_3_-on-reduced graphene oxide (MoS_3_-on-rGO) (Figure 4f), which exhibited a superior compatibility in KIBs. The contact angle tests (Figure 4g,h) showed that the amorphous MoS_3_-on-rGO electrode was endowed with a superior wetting property to the carbonate electrolyte owning to higher surface free energy of amorphous MoS_3_ and unique 3D interconnected porous structure, contributing to an optimized ion diffusion kinetics during the electrochemical process. When applied as the anode for KIBs, it exhibits high specific capacity (541 mAh g^−1^ at a current density of 0.2 A g^−1^) and excellent long-term cycling stability, which is significantly superior to the corresponding crystal sample (Figure 4i,j) [53]. Ji et al. took full advantage of element interaction to obtain phosphorus-doped amorphous carbon nanosheet (P-CNS) through thermal treatment, which achieved high performance as anode material for SIB. The long cycle stability exhibited a high specific capacity of 149 mAh g^−1^ for 5000 cycles at 5 A g^−1^ [54]. Amorphous FeO_x_ nanosheets with loose packing characteristic was developed by Hong and co-workers, which showed high electrochemical performance that specific capacity can be maintained at 263.4 mAh g^−1^ as an anode material for SIBs [55]. Bao et al. obtained a promising cathode material of SIB through coating amorphous FePO_4_ nanosheets on carbon nanosphere, which displayed high initial discharge capacity of 126.4 mAh g^−1^ at a current density of 20 mA g^−1^ and superior cycling performance [56].

### 3.2. Supercapacitor

**Figure 5:**
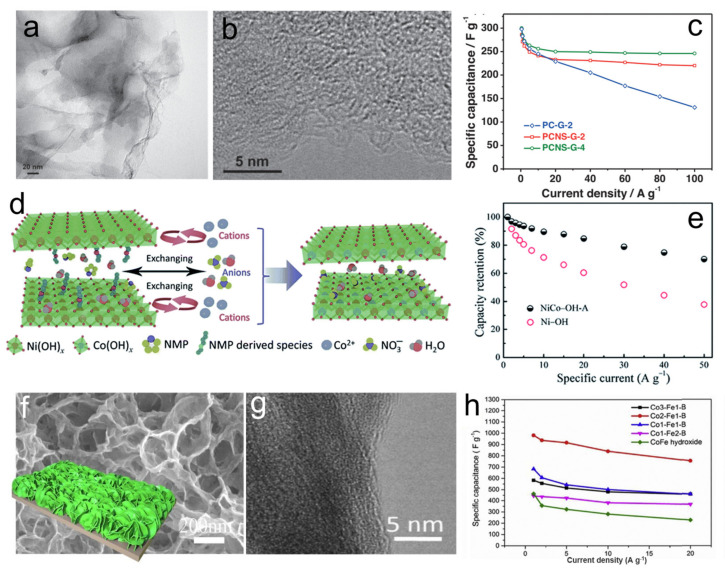
(**a**) TEM image of 2D porous carbon nanosheets; (**b**) HRTEM image of 2D porous carbon nanosheets; (**c**) Specific capacitances of 2D porous carbon nanosheets at different current densities. Reprinted with permission from Ref. [57]. Copyright Wiley-VCH, 2015. (**d**) Schematic diagram of the mechanism for ion exchange reactions; (**e**) Rate performance of amorphous Ni–Co hydroxide nanosheets. Reprinted with permission from Ref. [58]. Copyright Royal Society of Chemistry, 2018. (**f**) FESEM image and schematic illustration of the microstructure of NiCoMn–OH on Ni foams. Reprinted with permission from Ref. [60]. Copyright Elsevier, 2019. (**g**) HRTEM of the Co_0.2_Ni_0.8_ pyrophosphate nanosheets. (**h**) The cycling performance at the current density of 1.5 A g^−1^ for the Co_0.2_Ni_0.8_ pyrophosphate and Co_0.2_Ni_0.8_NH_4_PO_4_·H_2_O precursor. Reprinted with permission from Ref. [61]. Copyright Elsevier, 2019.

Supercapacitors (SCs) are being increasingly used to complement or partially replace batteries in various energy storage application owning to its high power density and exceptionally long lifetime. In the electrochemical reactions of SCs, the materials need to allow favorable diffusion of the electrolyte ions to access the active materials and cope with the strain and stress during charging/discharging process. Hence, manipulating 2D ANMs is now realized as an effective method to increase the electrochemical performance. The design of hole structure is considered to be one of the effective means to improve the performance of SCs. Qiu et al. developed a general approach for the synthesis of 2D porous carbon nanosheets from bio-sources-derived carbon precursors by an integrated procedure of intercalation, pyrolysis, and activation (Figure 5a,b). The as-prepared nanosheets possess optimized porous structures, which can shorten the ion transport distance during the charging/discharging process. When used as the electrode material in SCs, it shows a significantly improved rate performance with a high specific capacitance of 246 F g^−1^ and capacitance retention of 82% at 100 A g^−1^, being nearly twice than that of carbon particulates directly obtained from gelatin (Figure 5c) [57]. In addition, the synergistic interaction between different elements is also very effective in improving the performance of 2D ANMs in SC. Chen et al. synthesized Ni–OH nanosheets via a one-pot hydrothermal method. Then, a cation exchange reaction was conducted to exchange amorphous Ni(OH)_2_ with metal ions (Co^2+^, Mn^2+^, Cu^2+^ and Zn^2+^) to obtain a series of bimetal nanosheets (Figure 5d). Due to the higher activity from the combined contribution of Ni and Co, the NiCo–OH nanosheets show a superior specific capacity, rate performance, and cycling stability compared to that of pure Ni(OH)_2_ nanosheets (Figure 5e) [58]. Zhang et al. explored amorphous Co–Ni pyrophosphates nanosheets through controllably adjusting the ratios of Co and Ni. The optimized amorphous Ni–Co pyrophosphate showed much higher specific capacitance than monometallic Ni and Co pyrophosphates and exhibits excellent cycling ability [59]. Chen et al. proposed hydrothermal synthesis strategy to prepare amorphous NiCoMn–OH nanosheets, which was used as positive electrode materials for SC. The strong synergy between the transition metal ions in amorphous NiCoMn–OH is deemed to significantly promote the electrochemical activity, rate capability, and cycling stability. It is worth mentioning that the robust synthesis method was also used to fabricate the NiCoMn–OH porous network on conductive Ni foam (Figure 5f) and showed a specific capacity close to its theoretical value, indicating a complete utilization of the electroactive material [60]. Similarly, Zhu et al. fabricated ultrathin amorphous Co–Fe–B nanosheets on the 3D nickel foam substrate (Figure 5g) and the obtained sample showed an excellent specific capacitance (981 F g^−1^ at the 1 A g^−1^) and superior rate performance (Figure 5h) [61].

## 4. Manipulating 2D ANMs for Electrocatalysis

To build a clean future, massive efforts are underway to achieve high-efficiency and high-selectivity electrocatalysis systems for utilizing renewable energy and producing higher-value chemicals. Most typical electrocatalysis processes include nitrogen reduction reaction (NRR), carbon dioxide reduction reaction (CRR), oxygen reduction reaction (ORR) and water splitting which involves anodic oxygen evolution reaction (OER) and cathodic hydrogen evolution reaction (HER), etc. Catalysts play core role in diminishing energy loss and optimizing kinetics in these processes. Designing catalytic materials into 2D amorphous structure should be a wise and promising way since it can realize superiority combination of extended exposed area, abundant active sites, tunable electron states, and faster ion transport capacity. Accordingly, a batch of 2D ANMs have been developed to satisfy the urgent need. Here, we provide collective knowledge of manipulating 2D ANMs for electrocatalysis based on catalytic processes.

### 4.1. Water Splitting

Electrochemical water splitting is generally deemed as one of the most convenient and promising strategies to transform intermittent energy (e.g., solar and wind) to produce hydrogen. HER and OER are two- and four-electron processes, respectively, and thus OER is more kinetically sluggish and suffers from higher overpotentials. Traditional catalysts such as Pt or IrO_2,_ etc. with high activity are scarce and high cost. Some well-manipulated 2D ANMs have been demonstrated to possess satisfactory performance and compelling potential to substitute traditional noble metal catalysts.

**Figure 6:**
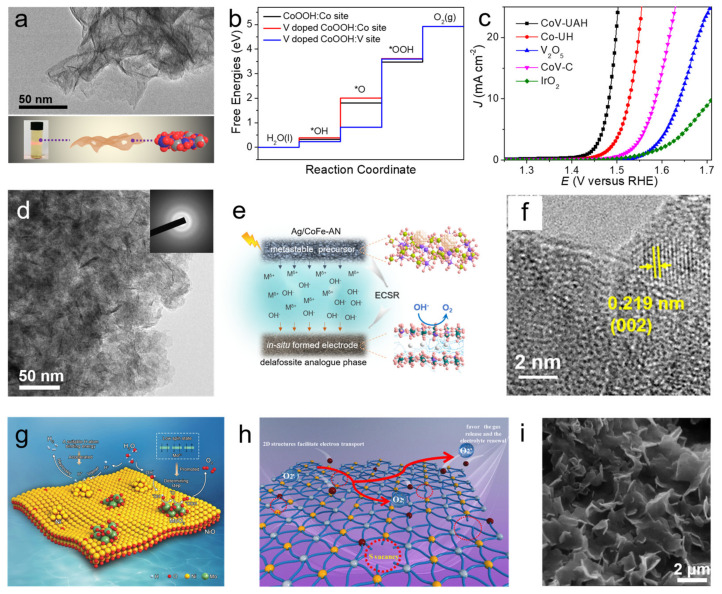
(**a**) The TEM image of CoV-UAH; (**b**) The free-energy landscape for V doped cobalt oxyhydroxide; (**c**) Linear sweep voltammetry curves for CoV-UAH and counterparts. Reprinted with permission from Ref. [17]. Copyright Royal Society of Chemistry, 2018. (**d**) The TEM image of amorphous delafossite analogue nanosheets, and inset is selected area electron diffraction (SAED); (**e**) Self-reconstruction process. Reprinted with permission from Ref. [62]. (**f**) HRTEM image of EBP/CoFeB sample. Reprinted with permission from Ref. [41]. Copyright American Chemical Society, 2021. (**g**) The enhanced mechanism of hybrid nanocatalysts for overall water splitting. Reprinted with permission from Ref. [72]. Copyright Wiley-VCH, 2020. (**h**) Activity mechanism for Mo–FeS nanosheets with sulfur defects. Reprinted with permission from Ref. [84]. Copyright American Chemical Society, 2020. (**i**) High-magnification SEM image of amorphous FePO_4_ nanosheets on Ni foam. Reprinted with permission from Ref. [88]. Copyright Wiley-VCH, 2017.

Tuning composition or introducing hetero atoms for element interaction should be an effective way to develop efficient 2D amorphous catalytic materials. Based on this modification strategy, Guo and co-workers developed a simple, yet robust one-step coprecipitation method to fabricate ultrathin amorphous cobalt-vanadium hydr(oxy)oxide nanosheets (CoV-UAH) with a thickness of ~0.7 nm (Figure 6a) [17]. The involvement of V is proved to give rise to the formation of ultrathin amorphous structure, which allows facile transformation to the desirable active phase consisting of V-doped cobalt oxyhydroxide species. First-principle simulations suggest that V doping can optimize reaction free energies of neighboring Co sites, leading to a theoretical low overpotential (Figure 6b). Thus, the talented material possesses large electrochemically active surface areas, low charge-transfer resistance, and impressive performance for the OER with a low overpotential of 0.250 V at a current density of 10 mA cm^−2^ and Tafel slope of 44 mV dec^−1^ superior to counterparts and commercial IrO_2_ (Figure 6c). In their other work, amorphous delafossite analogue nanosheets were fabricated by in situ electrochemical self-reconstruction, which features special structure of Ag intercalated into bimetallic cobalt-iron (oxyhydr)oxide layers (Figure 6d,e) [62]. The introduction of Ag modulates can regulate integral electron state and optimize electrocatalysis energetics, leading to superior OER activity and stability. Haik and co-workers doped Ga into amorphous cobalt boride nanosheets on Au support for smoothing growth of nanosheets and modifying surface electronic structure, thereby achieving a well-performed electrode with 230 mV overpotential to attain a current density of 10 mA cm^−2^ [63]. In another case, Co ion-intercalation can tune the structure of amorphous cobalt manganese oxide nanosheets at the atomic-scale to expose more active sites and allow easy penetration of electrolyte ions [64]. In HER field, highly active amorphous CoMoS4 nanosheets were constructed by coupling Mo to cobalt-based nanosheets, which showed favorable free energy change for H* adsorption and remarkable activity [65]. Some other metals can be benign dopants, such as Fe [38,39], Mo [66], Ag [62], etc. [67]. Surely, coupling nonmetal with 2D ANMs should also bring up beneficial synergistic effect. It is found that phosphating of metal (hydr)oxides (e.g., CoFe hydroxide, FeMnO_x_) nanosheets can result in amorphization so as to obtain highly active 2D amorphous catalyst for water splitting including both HER and OER with optimized catalytic sites and faster electronic transport [68,69]. Amorphous CoBP ternary alloy nanosheets were demonstrated to a well-performed HER catalyst [70]. Its remarkable activity can be attributed to synergistic effect of elements P and B, which accelerates dissociation of H_2_O, weakens surface H absorption, and suppresses Co oxidation.

In addition to element interaction, compositing hetero phases or substrates with 2D amorphous nanomaterials should also be a promising manner to realize multi-advantage integration. Chen et al. constructed a 2D heterostructure of EBP/CoFeB, which exhibits excellent OER activity with an ultralow overpotential of 227 mV at 10 mA cm^−2^ and excellent stability (Figure 6f) [41]. This nanohybrid structure not only optimizes the reactive intermediate absorption, but also combines the high conductivity of black phosphorus. Combining amorphous phase with crystalline phase also seems to be a wise way to promote catalytic properties of catalysts. Huang and co-workers rationally designed channel-rich RuCu nanosheets composed of crystallized Ru and amorphous Cu for overall water splitting in pH-universal electrolytes [71]. The amorphous/crystalline compositing structure is endowed with highly active electron transfer and optimized electronic structures. Furthermore, Hu and co-workers developed amorphous NiO nanosheets coupled with crystalline Ni and MoO_3_ nanoparticles, which exhibited two heterostructures of Ni–NiO and MoO_3_–NiO (Figure 6g) [72]. The deliberately manipulated structure dramatically diminishes the energetic barrier and works as catalytically active centers, synergistically improving the overall water splitting. The similar strategy was also adopted to develop crystalline platinum oxide-decorated amorphous cobalt oxide hydroxide nanosheets and amorphous RuCu nanosheets grown on crystalline Cu nanotubes as HER catalysts [46,73]. Besides, enhanced catalytic effect by compositing hetero phases can be achieved by assembling amorphous nanosheets with nanodots [74], carbon nanofibers [75], metal oxide [76,77,78], etc. [79,80]. It should be mentioned that anchoring amorphous nanosheets on high conductivity substrate should be an effective way to afford electrolysis at large current density. Zhao and co-worker electrodeposited amorphous mesoporous nickel-iron composite nanosheets directly onto macro-porous nickel foam as OER electrode, which can deliver current densities of 500 and 1000 mA cm^−2^ at overpotentials of 240 and 270 mV, respectively [42]. Zhang et al. anchored amorphous MoS_2_ nanosheet arrays on carbon cloth to form a three-dimensional nanostructure with abundant exposed edge sites [81]. The composite exhibited satisfactory catalytic activity and durability for the HER in acidic solutions. In addition, Ni foil [82], graphite foil [44], and Ti plate [83] were also proved to be good substrates.

Defect design and porousness manipulation should be regarded as micro-design on geometric configuration, which can be easily carried out on 2D amorphous structure. Shao et al. found sulfur defects could modulate the electron state and Gibbs free energies of amorphous Mo–FeS nanosheets, leading to preferable OER kinetics (Figure 6h) [84]. Mo defects could be created on monolayer amorphous molybdenum sulfide and identified as catalytically active sites for HER [37]. In addition, porousness and channel design also show positive effect for electrocatalysis of water splitting [71]. Amorphous cobalt phosphate nanosheets, amorphous CoS_x_(OH)_y_ nanosheets, and amorphous NiCoFe phosphate nanosheets with well-designed porous structure were developed as efficient OER catalysts [85,86,87]. The porous characteristic can provide large free space, increased distribution of the active centers, and facile movement of reactants and products, thus enhanced catalysis performance can be achieved. Except for micro-design, the spatial arrangement of amorphous nanosheet should be noteworthy. Yang et al. realized in situ vertical growth of amorphous FePO_4_ nanosheets (Figure 6i) on Ni foam which demonstrated excellent catalytical activity and stability in overall water splitting [88]. This type of geometric configuration is favorable to electron transport, electrolyte diffusion, and structural stability in catalysis processes.

### 4.2. Electrochemical Reduction Reactions

**Figure 7:**
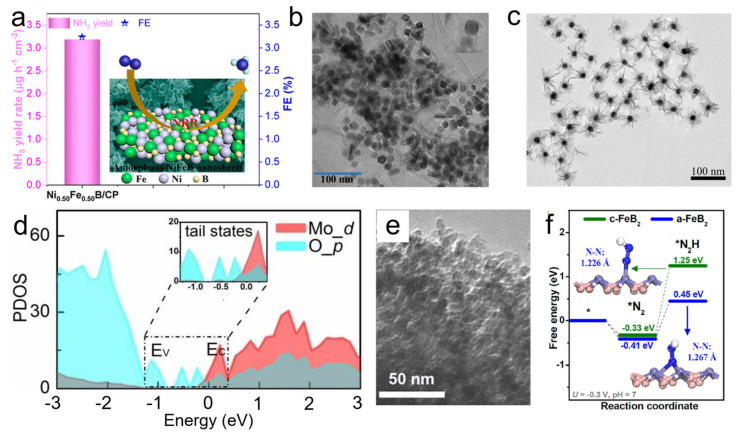
(**a**) Schematic diagram of NRR on amorphous NiFeB nanosheets. Reprinted with permission from Ref. [89]. Copyright American Chemical Society, 2020. (**b**) TEM image of PdH_x_@Ni–B. Reprinted with permission from Ref. [93]. Copyright Wiley-VCH, 2017. (**c**) TEM image of Au nanocrystals@amorphous MnO_2_ nanosheets. Reprinted with permission from Ref. [94]. Copyright American Chemical Society, 2021. (**d**) Anderson tail states of amorphous MoO_3-x_. Reprinted with permission from Ref. [95]. Copyright Wiley-VCH, 2019. (**e**) TEM image of porous FeB_2_ nanosheets. (**f**) Free energy diagrams of *N_2_ and *N_2_H adsorption on crystalline FeB_2_ and amorphous FeB_2_. Reprinted with permission from Ref. [97]. Copyright Elsevier, 2021.

Some 2D ANMs were also manipulated to catalyze electrochemical reduction reactions, such as ORR, CRR, and NRR. As to positive effect induced by component interaction for electrochemical reduction reactions, similar ways of tuning composition or introducing hetero atoms and compositing hetero phases or substrates are also advisable. Wang et al. tailored amorphous NiFeB nanosheets for enhancing the electrocatalytic NRR kinetics by adjusting the ratio of Ni/Fe/B (Figure 7a) [89]. The collective merits of amorphous structure and optimized element ratio gives rise to synergistic effect in creating active sites and facilitating the N_2_ adsorption capacity, thereby leading to superior NRR activity with a high NH_3_ formation rate of 3.24 μg h^−1^ cm^−2^. Sun and co-workers developed biomass-derived oxygen-doped amorphous carbon nanosheets with high electrochemical selectivity and activity for NRR [90]. Similarly, highly efficient ORR catalysts with abundant active sites and high conductivity were fabricated by doping N or P into amorphous carbon sheets [91,92]. As mentioned above, amorphous nanosheets possess flexible morphology and structure, which can provide an ideal platform for supporting or embedding nanoparticles. In a work by Lu et al., a unique hybrid catalyst of Pd hydride nanocubes encapsulated within amorphous Ni–B nanosheets (PdH_x_@Ni–B) was synthesized and demonstrated an impressive ORR activity (1.05 mg_Pd_^−1^ at 0.90 V versus reversible hydrogen electrode) and stability (10,000 potential cycles) (Figure 7b) [93]. Gao et al. constructed a composite catalyst of Au nanocrystals@amorphous MnO_2_ nanosheets with CO faradic efficiency (FE) of 90.5% for CRR at −0.7 V versus reversible hydrogen electrode [94]. The core/shell nanostructure brings about the interaction between Au and amorphous MnO_2_ nanosheets, which contributed to its performance (Figure 7c). Specially, Yuan et al. reported the mass-production of amorphous SnO_x_ nanoflakes modified by BiO_x_ species from nanoparticles to single atoms, which exhibited an FE of HCOOH over 90% in CRR [20].

For electrochemical reduction reactions, the optimization of geometric configuration is also recommendable. As typical cases, amorphous MoO_3-x_ monolayers with oxygen vacancies and amorphous MoS_3_ nanosheets with sulfur vacancies can work as efficient NRR catalysts [95,96]. The vacancy defects are able to modulate electron state of catalysts and reduce energetics barriers for facilitating NRR process and simultaneously suppressing HER (Figure 7d). Additionally, porous design is achieved on amorphized FeB_2_ nanosheets for boosted NRR activity with an NH_3_ yield of 39.8 μg h^−1^ mg^−1^ (Figure 7e) [97]. The porous amorphous structure could upraise the d-band center of a-FeB_2_ and strengthen the absorption of key *N_2_H intermediate, thereby reducing reaction barrier (Figure 7f).

## 5. Conclusions and Outlook

The interests in 2D ANMs are growing continuously along with the extensive study of amorphous material science. These materials are promising candidates for facilitating the key processes of electrochemical energy storage and conversion systems due to their unique advantages of large specific surface area, excellent “in-plane” charge-carrier transport, abundant defects, etc. However, some issues still exist for their electrochemical application: (i) synthesis systems and mechanisms are lacking and ambiguous, which limit their categories and quantity production; (ii) most of the synthesized 2D ANMs are metal oxide with poor conductivity; (iii) the dispersity and structural stability of 2D ANMs are unsatisfactory due to the high surface energy; (iv) intrinsic activity still deserves improvement. Hence, it is indeed necessary to explore more effective and reliable methods to optimize this family of materials for preferable electrochemical application.

In this review, we summarized effective strategies to manipulate on 2D ANMs and their applications in battery, supercapacitor, and electrocatalysis. We conceptualized these strategies to be geometric configuration design and component interaction, concretely embodying in spatial structure and coordination environment design as well as elemental interaction and heterophase compositing. For geometric configuration, the introduction of pores or defects within nanosheets can provide more active sites, superior electrolyte diffusion and ion transport kinetics. As to component interaction, heteroatom doping can change the band structure and electronic properties, while heterophase compositing enable advantage integration to achieve improved conductivity and stability. Thanks to the flexible structure of 2D ANMs, these elaborate manipulations can be realized by deliberate synthetic routes. These manipulated 2D ANMs with optimized structures and properties demonstrated enhanced electrochemical performance, while discriminatory manipulation ways are related with different applications.

2D ANMs are intriguing, and manipulating them for purposive application is promising. Despite the visible progress that has been witnessed, there are still many issues to be addressed: i) 2D amorphous structure is mysterious, which retards our deeper cognition; ii) immature synthesis methods; iii) controllable manipulation means are still lacking, especially at atomic scale; iv) in-depth understanding to the roles of well-built structure in electrochemical processes is insufficient. To meet these challenges, more advanced characterization techniques are needed to clarify the nature of 2D amorphous structures, formation mechanisms, and functional rules. Meanwhile, some experience can be selectively drawn from crystalline systems. As such, the discovery, manipulation, and application of 2D ANMs following the success of amorphous materials have opened up a new pave for sustainable energy applications. It is believed that the development of new 2D ANMs and their derived materials will further not only play a role in improving the performance of sustainable energy devices and contribute to resolving the current environmental and energy crises, but also stimulate the advances in amorphous science field.

## Data Availability

Not applicable.
